# Proteomic Profiling Identifies Kaposi’s Sarcoma-Associated Herpesvirus (KSHV)-Encoded LANA^SIM^-Associated Proteins in Hypoxia

**DOI:** 10.1128/mSystems.01109-21

**Published:** 2021-11-02

**Authors:** Xiaoqing Liu, Jin Gan, Shujuan Du, Caixia Zhu, Yuyan Wang, Yuping Jia, Daizhou Zhang, Di Qu, Fang Wei, Erle S. Robertson, Qiliang Cai

**Affiliations:** a Shanghai Institute of Infections Disease and Biosecurity & MOE&NHC&CAMS Key Laboratory of Medical Molecular Virology, Department of Medical Microbiology and Parasitology, School of Basic Medicine, Shanghai Medical College, Fudan University, Shanghai, People’s Republic of China; b ShengYushou Center of Cell Biology and Immunology, School of Life Sciences and Biotechnology, Shanghai Jiao Tong University, Shanghai, People’s Republic of China; c Shandong Academy of Pharmaceutical Sciences, Jinan, People’s Republic of China; d Department of Microbiology, Perelman School of Medicine at the University of Pennsylvania, Philadelphia, Pennsylvania, USA; e Abramson Comprehensive Cancer Center, Perelman School of Medicine at the University of Pennsylvania, Philadelphia, Pennsylvania, USA; f Expert Workstation, Baoji Central Hospital, Baoji, People’s Republic of China; Princeton University

**Keywords:** KSHV, LANA, SIM, hypoxia, HNRNPU, SUMO-interacting motif

## Abstract

Hypoxia signaling is a key regulator in the development and progression of many types of human malignancies, including viral cancers. The latency-associated nuclear antigen (LANA), encoded by Kaposi’s sarcoma-associated herpesvirus (KSHV) during latency, is a multifunctional protein that plays an essential role in viral episome maintenance and lytic gene silencing for inducing tumorigenesis. Although our previous studies have shown that LANA contains a SUMO-interacting motif (LANA^SIM^), and hypoxia reduces SUMOylated KAP1 association with LANA^SIM^, the physiological proteomic network of LANA^SIM^-associated cellular proteins in response to hypoxia is still unclear. In this study, we individually established cell lines stably expressing wild-type LANA (LANA^WT^) and its SIM-deleted mutant (LANA^dSIM^) and treated them with or without hypoxia, followed by coimmunoprecipitation and mass spectrometry analysis to systemically identify the hypoxia-responsive profile of LANA^SIM^-associated cellular proteins. We found that in hypoxia, the number of cellular proteins associated with LANA^WT^ instead of LANA^dSIM^ was dramatically increased. Functional network analysis revealed that two major pathways, which included cytoskeleton organization and DNA/RNA binding and processing pathways, were significantly enriched for 28 LANA^SIM^-associated proteins in response to hypoxia. HNRNPU was one of the proteins consistently identified that interacted with LANA^SIM^ in different proteomic screening systems and responded to hypoxia. This study provides a proteomic profile of LANA^SIM^-associated proteins in hypoxia and facilitates our understanding of the role of the collaboration between viral infection and the hypoxia response in inducing viral persistence and tumorigenesis.

**IMPORTANCE** Kaposi’s sarcoma-associated herpesvirus (KSHV) has been reported to be involved in the regulation of host proteins in response to hypoxic stress. LANA, one of the key latent proteins, contains a SUMO-interacting motif (LANA^SIM^) and reduces the association with SUMOylated KAP1 upon hypoxic treatment. However, the physiological systematic network of LANA^SIM^-associated cellular proteins in hypoxia is still unclear. Here, we revealed two major pathways, which included cytoskeleton organization and DNA/RNA binding and processing pathways, that were significantly enriched for 28 LANA^SIM^-associated proteins in hypoxia. This discovery not only provides a proteomic profile of LANA^SIM^-associated proteins in hypoxia but also facilitates our understanding of the collaboration between viral infection and hypoxic stress in inducing viral persistence and tumorigenesis.

## INTRODUCTION

Hypoxia (low oxygen) is a common feature of the hostile microenvironment that results from the rapid growth of cancer cells (1). It has been demonstrated that hypoxia occurs in many types of human malignancies, including virus-mediated cancers (2). Kaposi’s sarcoma (KS)-associated herpesvirus (KSHV), or human herpesvirus 8 (HHV-8), mainly infects two cell types, human endothelial cells and B lymphocytes, and is tightly associated with KS, primary effusion lymphoma (PEL), and a subset of multicentric Castleman’s diseases (MCDs) (3). Like other herpesviruses, KSHV has two stages in its life cycle: latent infection and lytic replication. During latency, the KSHV genome is anchored to the host chromosome as an episome, with only a few viral genes being expressed (4). The latency-associated nuclear antigen (LANA), encoded by open reading frame 73 (ORF73), is one of the viral proteins dominantly expressed in latent infection. It has been demonstrated that LANA is a nuclear protein with multiple functions, particularly tethering the viral episome to host chromatin by acting as an adaptor (through linking the tandem repeat [TR] region of the viral genome with histones H1 and H2A/B of host chromosomes via binding to its C terminus and N terminus, respectively) to ensure the maintenance of the viral genome during host cell segregation (5). In addition, LANA can directly or indirectly interact with a number of host proteins, such as binding to the promoter region of host/viral genes and inhibiting viral lytic replication, host cell immune responses, and apoptosis, thereby promoting the establishment and maintenance of KSHV latent infection and ultimately inducing the formation of tumors (6). We and other groups have also shown that LANA not only interacts with HIF1α (one of the key hypoxia-inducible factors) to block lytic replication under normoxic conditions but also protects the cellular replication machinery from hypoxia-induced degradation (7, 8), although hypoxia could reactivate KSHV from latency to lytic replication (7). However, the molecular mechanisms of how KSHV controls its life cycle in response to hypoxia remain largely unclear.

Emerging evidence indicates that small ubiquitin-like modifier (SUMO), one of the posttranslational modifications of proteins, plays a critical role in the regulation of gene transcription and the response to extracellular stress (9, 10). In addition to covalently attaching to substrate proteins, SUMO can also noncovalently bind to target proteins through a SUMO-interacting motif (SIM), which contains a consensus sequence including K-x_3–5_-I/V-I/L-I/L-x_3_-D/E/Q/N-D/E-D/E, h-h-x-S-x-S/T-a-a, or V/I-x-V/I-V/I (11–13). Our previous work showed that there is a SIM localized within the amino terminus of the LANA protein (LANA^SIM^), which can recruit a variety of proteins, including SUMO2-modified KAP1 and Sin3A, to form a transcription-inhibitory complex. This complex in turn silences gene expression and maintains the viral episome along with host cell segregation during latency. In hypoxia, the SUMOylated protein, including KAP1, is dissociated from the LANA^SIM^-associated complex, which opens the chromatin structure to activate viral lytic replication (14). By expressing and purifying the recombinant protein of the SIM of LANA fused with glutathione *S*-transferase (GST) as bait *in vitro* to capture the LANA^SIM^-interacting proteins from nuclear extracts of PEL cells, combined with mass spectrometry (MS) analysis, we identified a series of proteins that are mainly involved in the regulation of the cell cycle, DNA unwinding and replication, and pre-mRNA/mRNA processing associated with LANA^SIM^ (15). However, a limitation of the previous study was that only amino acids 240 to 300 of LANA were used for capturing interacting proteins; this short amino acid sequence might not be representative of the structure of LANA^SIM^, and *in vitro* GST pulldown assays could not fully reflect the endogenous protein-protein interactions that physiologically exist within the cellular complexes in cells.

In this study, to further elucidate the physiological functions of LANA^SIM^ in response to hypoxia, we initially generated human umbilical vein endothelial cell (HUVEC) and HEK293 cell lines stably expressing wild-type LANA (LANA^WT^) and its SIM-deleted mutant (LANA^dSIM^) individually, followed by treatment with or without hypoxia. We then performed coimmunoprecipitation combined with tandem quantitative mass spectrometry analyses to identify and highlight the interacting proteomic profile of LANA^SIM^ in response to hypoxia. These results provide new insights into the collaborative role of LANA^SIM^-mediated proteins between viral infection and the hypoxia response for viral pathogenesis and tumorigenesis.

## RESULTS

### Quantitative proteomic analysis of LANA^SIM^-associated proteins in hypoxia.

**FIG 1:**
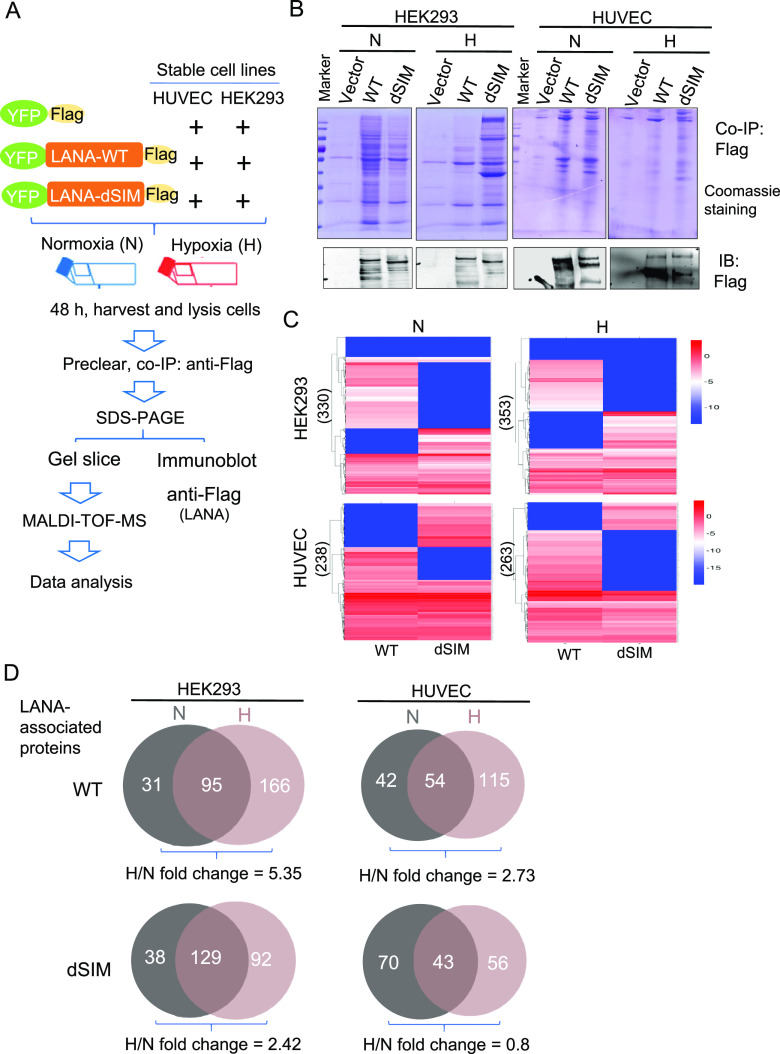
Overview of the strategy to identify the hypoxia-sensitive proteins that interact with LANA^SIM^. (A) Streamlined workflow for identification of hypoxia-sensitive proteins that interact with LANA^SIM^ as determined by proteomic analysis. HUVECs or HEK293 cells stably expressing YFP-Flag, YFP-LANA^WT^-Flag, or YFP-LANA^dSIM^-Flag were cultured in normoxia (N) (21% O_2_) or hypoxia (H) (0.2% O_2_) for 48 h. Total protein extracts from these cells were then subjected to coimmunoprecipitation (co-IP) with anti-Flag antibodies, and the precipitated proteins were separated on SDS-PAGE gels. Following in-gel trypsin digestion, peptides were isolated and purified for MALDI-TOF MS analysis or immunoblotting with anti-Flag antibodies for confirming the levels of exogenous LANA expression. (B) Coomassie staining of SDS-PAGE gels for separation of precipitated YFP-Flag, LANA^WT^-Flag, or LANA^dSIM^-Flag proteins from HUVEC or HEK293 stable cell lines in the presence or absence of hypoxia treatment as described above for panel A. The levels of exogenous LANA from immunoblot analysis with anti-Flag antibodies are shown in the bottom panels. (C) Heat map showing the number of proteins associated with LANA^WT^ or LANA^dSIM^ in HUVEC and HEK293 stable cell lines in normoxia or hypoxia. The color gradient represents the log_2_ exponentially modified protein abundance index (emPAI) values after excluding the background of the vector group as a control. (D) Venn diagrams of proteins interacting with LANA^WT^ (top) or LANA^dSIM^ (bottom) from HUVEC and HEK293 stable cell lines in the presence or absence of hypoxia identified by MS. The number is obtained based on peptides of the proteins detected from the mass spectrum with the exclusion of nonspecifically binding proteins. The ratios of the interacting proteins of LANA^WT^ (or LANA^dSIM^) under normoxic and those under hypoxic conditions are shown at the bottom.

To identify proteins specifically associated with the SIM of LANA (LANA^SIM^) in a physiological setting (Fig. 1A), we generated two types of cell lines (HUVECs and HEK293 cells) stably expressing recombinant wild-type LANA fused with both yellow fluorescent protein (YFP) and Flag tags (YFP-LANA^WT^-Flag), its SIM-deleted mutant (YFP-LANA^dSIM^-Flag), or the vector alone (YFP-Flag). The stable cell lines were then individually subjected to normoxia or hypoxia treatment for 48 h, followed by coimmunoprecipitation assays with anti-Flag antibodies and relative quantitative matrix-assisted laser desorption ionization–time of flight (MALDI-TOF) MS analysis and protein sequence identification (Fig. 1A). As shown in Fig. 1B, along with immunoblot (IB) analysis to verify the expression level of LANA as a control, we observed that the bands representing the interacting cellular proteins were enriched by LANA^WT^ or LANA^dSIM^ in both HUVEC and HEK293 stable cell lines, with both hypoxia and normoxia treatments, compared with the vector-alone group (Fig. 1B, top). Interestingly, the deletion of the SIM of LANA or hypoxia treatment consistently reduced the intensity of the interacting proteins pulled down by LANA in both HUVEC and HEK293 stable cell lines, which supported our previous observation that LANA^SIM^ functions in response to hypoxic stress (14). To confirm whether the LANA^SIM^-interacting proteins present higher sensitivity in response to hypoxia, the immunoprecipitated complexes of LANA^WT^ and LANA^dSIM^ from both HUVEC and HEK293 stable cell lines in normoxia or hypoxia were individually subjected to MALDI-TOF MS analysis (Fig. 1C), along with the vector alone as a parallel control to exclude the nonspecifically binding proteins. The results showed that 96 cellular proteins interacted with LANA^WT^ from HUVECs in normoxia, while 169 cellular proteins were associated with hypoxia. Notably, 54 cellular proteins appeared in both normoxia and hypoxia (Fig. 1D, right). In contrast, despite the fact that 70 cellular proteins exclusively interacted with the SIM-deleted mutant of LANA (LANA^dSIM^) from HUVECs in normoxia, only 56 cellular proteins were associated exclusively with hypoxia. The number of cellular proteins pulled down by LANA^WT^ from stable HUVECs in hypoxia and normoxia was much higher (fold change for the WT versus dSIM of 2.73- versus 0.8-fold) than that pulled down by LANA^dSIM^ (Fig. 1D, right). Consistently, a similar phenomenon (fold change for the WT versus dSIM of 5.35- versus 2.42-fold) was observed in HEK293 stable cell lines (Fig. 1D, left). This indicates that the loss of the SIM may reduce the ability of LANA to deal with hypoxic stress.

**FIG 2:**
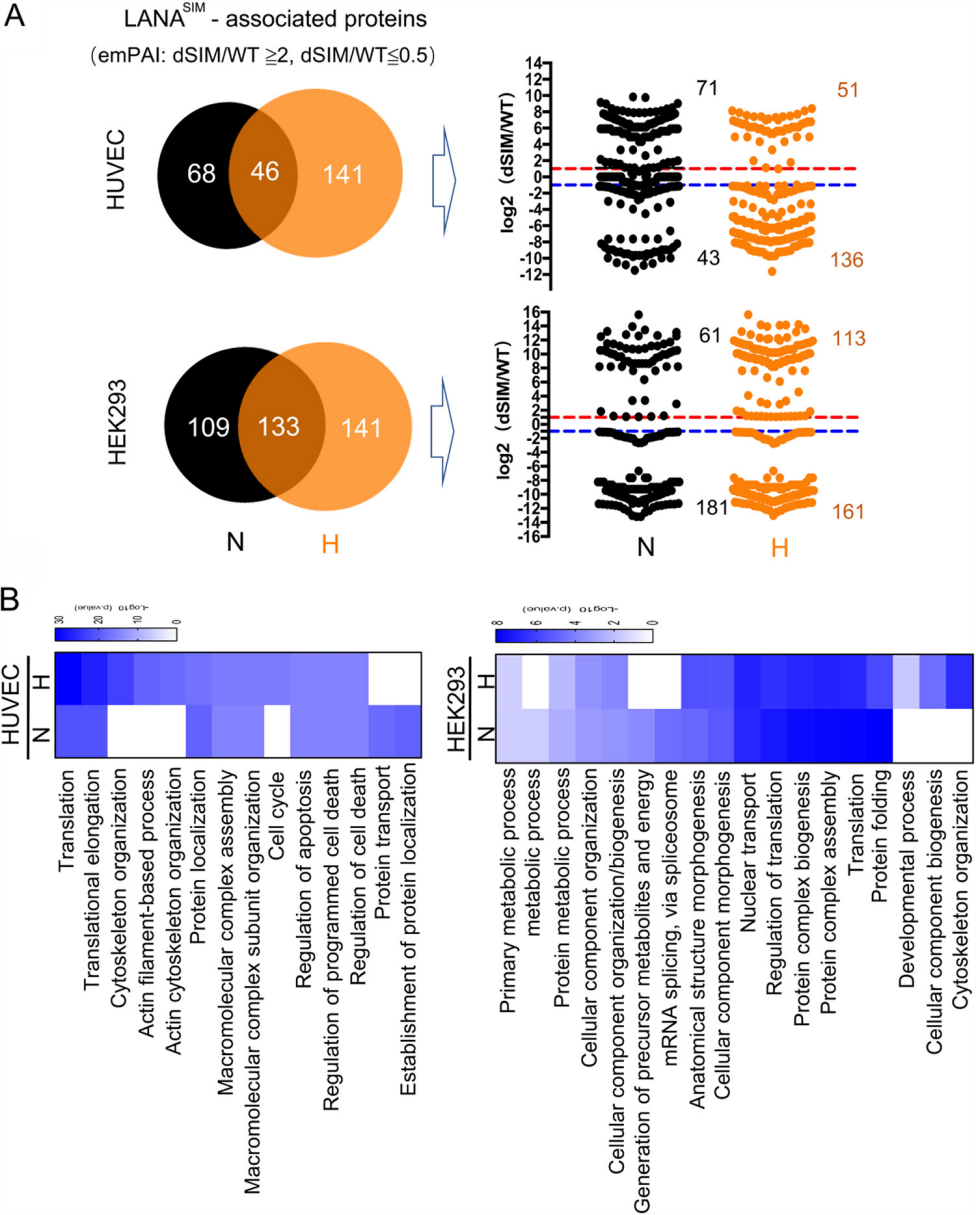
LANA^SIM^-associated protein profiles from HUVECs and HEK293 cells in response to hypoxia. (A) Venn diagrams of the identified LANA^SIM^-associated proteins from LANA^WT^- or LANA^dSIM^ stably expressing cells by mass spectrometry analysis based on an emPAI ratio of LANA^dSIM^/LANA^WT^ of ≥2 (increased with the deletion of SIM) or ≤0.5 (decreased with the deletion of SIM). The distribution data for each protein with log_2_ (dSIM/WT) values in normoxia (N) or hypoxia (H) are shown in the right panel. (B) Heat maps of significantly enriched biological pathways of LANA^SIM^-associated proteins identified from stable cells in normoxia or hypoxia from panel A. The color gradient from blue to white represents low to high *P* values.

To further explore the role of LANA^SIM^, we analyzed LANA^SIM^-associated protein profiles from HUVEC or HEK293 stable cell lines under normoxic or hypoxic conditions, based on the screening criterion of an emPAI ratio of LANA^dSIM^/LANA^WT^ of ≥2 (increased with the deletion of SIM) or ≤0.5 (decreased with the deletion of SIM). As shown in the top panels of Fig. 2A, there were 114 cellular proteins associated with LANA^SIM^ from stable HUVECs in normoxia, while there were 187 proteins in hypoxia. Among these, 71 proteins were increased and 43 were decreased in normoxia, and 51 proteins were increased and 136 were decreased in hypoxia. A similar phenomenon was also observed in the HEK293 stable cell lines (Fig. 2A, bottom). Functional clustering analysis revealed that LANA^SIM^-associated cellular proteins from stable HUVECs in hypoxia were enriched mainly in cell cycle and cytoskeleton organization pathways and less so for protein transport or protein localization pathways compared to what was seen in normoxia (Fig. 2B, left). In contrast, LANA^SIM^-associated cellular proteins from HEK293 stable cells in hypoxia were also predominantly enriched in the cytoskeleton organization pathway, in addition to the cellular component biogenesis pathway, and more limited in the association with proteins in the mRNA splicing/spliceosome and metabolic process pathways (Fig. 2B, right). This provides evidence that the key biological processes associated with the SIM of LANA are different in normoxia and hypoxia, and the cytoskeleton organization pathway may be a common target of LANA^SIM^ in hypoxia.

### Comparison between LANA^SIM^- and LANA^dSIM^-associated proteomic profiles in hypoxia.

**FIG 3:**
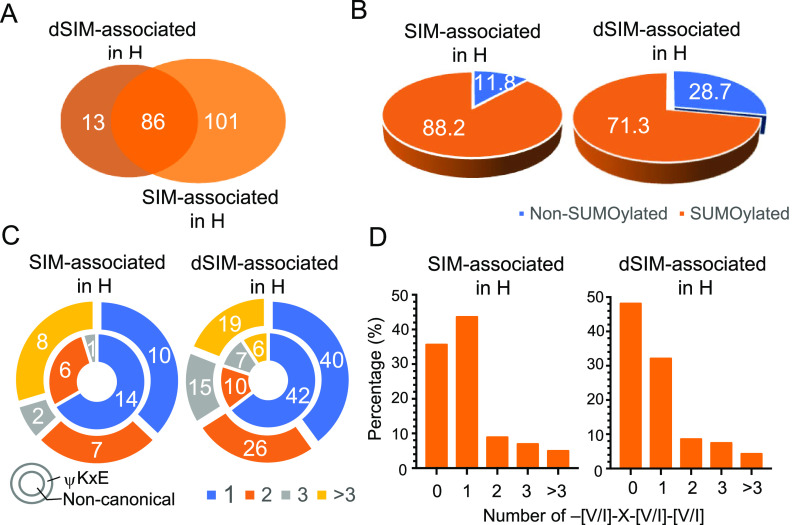
Differential proteome profiles of LANA^SIM^- and LANA^dSIM^-associated proteins in hypoxia. (A) Venn diagrams of the numbers of LANA^SIM^- and LANA^dSIM^-associated proteins from HUVECs in hypoxia identified by MS. (B) Relative percentages of SUMOylated and non-SUMOylated proteins associated with LANA^SIM^ or LANA^dSIM^ in hypoxia from panel A. The relative percentage was calculated according to the number of identified proteins containing the ΨKxE motif or noncanonical consensus SUMOylated sites, which was predicted by SUMOsp_2.0 with the high threshold setting. (C) Relative percentages of LANA^SIM^- or LANA^dSIM^-associated proteins with the ΨKxE motif (outer circle) or noncanonical consensus SUMOylated sites (inner circle). The different numbers (1, 2, 3, or >3) of ΨKxE motifs in each protein are shown in different colors at the bottom. (D) Relative percentages of the –[V/I]-X-[V/I]-[V/I]– consensus SIMs.

To validate whether those proteins enriched by LANA^SIM^ in hypoxia are related to SUMO modification, we chose HUVECs as a model for further analysis and compared the differences between LANA^SIM^-associated proteins and LANA^dSIM^-associated proteins. As described previously (15), we predicted the potential SUMO modification sites and SIMs from 101 (out of 187) proteins exclusively associated with LANA^SIM^ in hypoxia and 13 (out of 99) proteins exclusively associated with LANA^dSIM^ as a control (Fig. 3A). As shown in Fig. 3B, 88.2% of the identified proteins have a high potential for being SUMOylated in the LANA^SIM^-associated profile, while a relatively lower number of proteins (71.3%) was identified in the LANA^dSIM^-associated profile (Fig. 3B). Unexpectedly, the distributions of LANA^SIM^- and LANA^dSIM^-associated proteins with different numbers (1, 2, 3, or >3) of ΨKxE or noncanonical consensus SUMOylated sites were similar (Fig. 3C). The identified proteins containing only one potential SUMOylation site were the majority in both the LANA^SIM^- and LANA^dSIM^-associated profiles (Fig. 3C). Consistent with our previous study, the interaction of SUMOylated proteins with LANA^SIM^ has a higher capacity for SIMs, with about 48.2% of the LANA^SIM^-associated proteins in hypoxia but only 30.5% of the LANA^dSIM^-associated proteins containing at least 1 SIM (Fig. 3D). This indicates that the LANA^SIM^-associated proteins in hypoxia are more easily SUMOylated than the LANA^dSIM^-associated proteins in hypoxia.

### The functional core network of LANA^SIM^-associated proteins in hypoxia.

**FIG 4:**
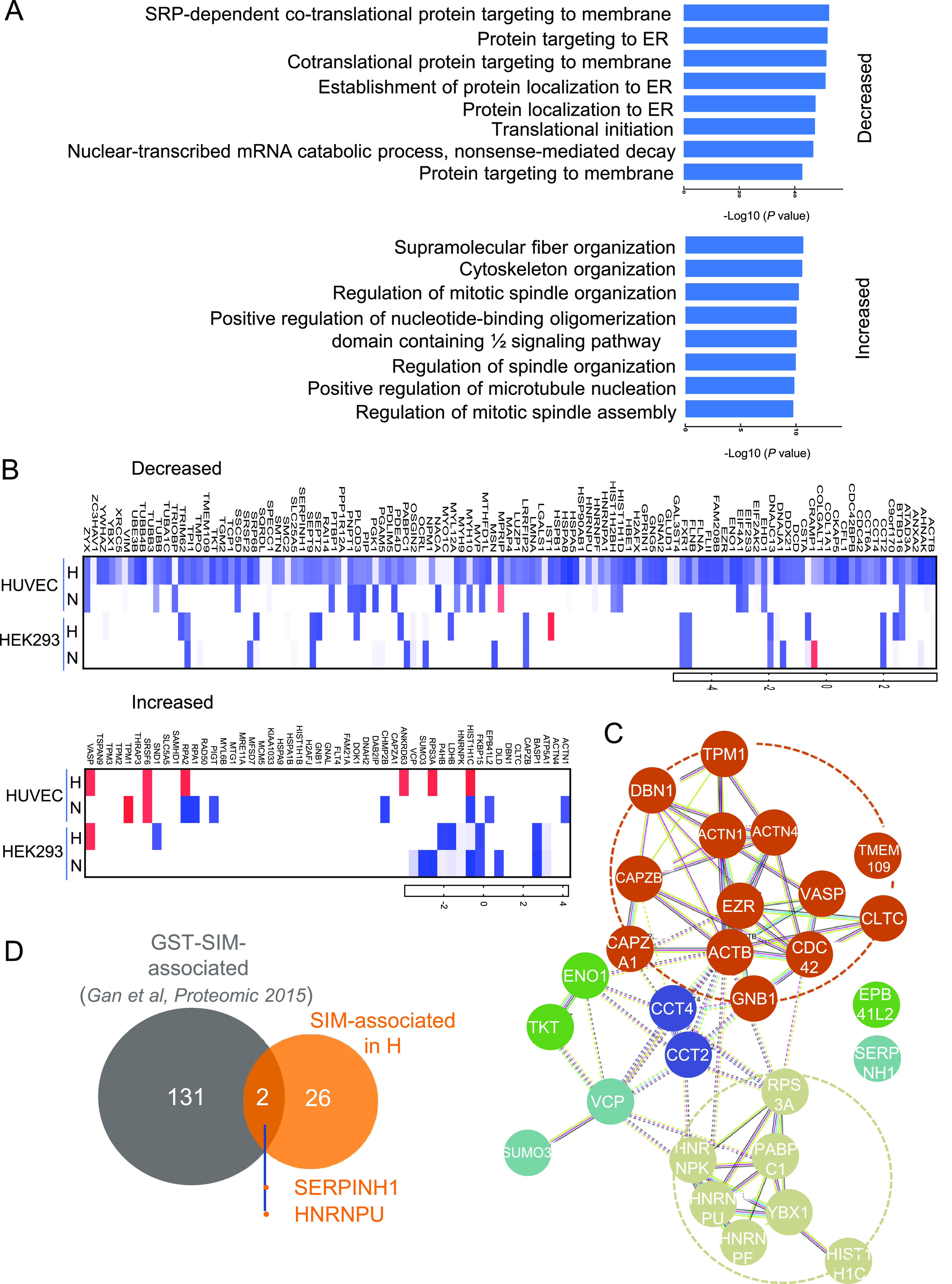
Functional profile analysis of the LANA^SIM^-associated proteins in response to hypoxia. (A) Biological pathway analyses of identified proteins with decreased or increased associations with LANA^SIM^ in HUVECs in response to hypoxia from Fig. 2A. The top 8 pathways for the enrichment of differentially expressed proteins are shown. ER, endoplasmic reticulum. (B) Heat map of identified proteins with significantly decreased or increased associations with LANA^SIM^ in both HUVECs and HEK293 cells under normoxia or hypoxia from Fig. 2A. The relative fold changes are shown as log ratios of emPAI values of dSIM/WT. (C) Hypothetical regulatory core network of 28 identified proteins with increased or decreased associations with LANA^SIM^ in both HUVECs and HEK293 cells in response to hypoxia. This analysis was performed using STRING (https://string-db.org). The red dotted circle indicates the molecules involved in the regulation of cytoskeleton organization and protein localization; DNA/RNA binding and processing are shown by the light green dotted circle. (D) Venn diagram of the numbers of LANA^SIM^-associated proteins from HUVECs/HEK293 cells in hypoxia and GST-SIM-associated proteins from BCBL1 cells identified by MS (15).

Since increased and decreased profiles of the LANA^SIM^-associated proteins from HEVECs or HEK293 cells in hypoxia were observed once the SIM was deleted (Fig. 2A, right), we attempted to perform an enrichment analysis of the related functional pathways targeted by LANA^SIM^. The results showed that functional pathways of the decreased proteins targeted by LANA^SIM^ in hypoxia were mainly related to protein localization, translational initiation, and mRNA processing, while the increased ones mainly participated in the regulation of cytoskeleton organization and spindle organization (Fig. 4A). To further evaluate the consistency of the increased and decreased profiles of the LANA^SIM^-associated proteins in hypoxia, we analyzed and aligned the LANA^SIM^-associated proteins from HUVECs and HEK293 cells in hypoxia (Fig. 4B) and found that 16 of the increased proteins and 12 of the decreased proteins were consistently associated with LANA^SIM^ (Table 1). Interestingly, the functional core network analysis of these 28 proteins revealed that the LANA^SIM^-associated proteins in hypoxia are mainly involved in the regulation of cytoskeleton organization and protein localization and DNA/RNA binding and processing (Fig. 4C and Table 1). Among these, SUMO3, a well-known SUMO molecule that responds to hypoxic stress, was also observed (Fig. 4C). This supports our hypothesis and previous reports from our group and other investigators (15–17).

**TABLE 1:** Characteristics of 28 LANA^SIM^-associated proteins identified from HUVECs and HEK293 cells in hypoxia

Gene symbol	Mass	Gene description	Log_2_ fold change (dSIM/WT)		Core pathway(s)
			HUVECs	HEK293 cells	
ACTB	41,710	Actin, cytoplasmic 1	−4.29	−0.24	Cytoskeleton organization
ACTN1	106,714	Alpha-actinin-1 isoform X1	3.28	4.26	Cytoskeleton organization
ACTN4	104,788	Alpha-actinin-4	2.85	1.84	Cytoskeleton organization
CAPZA1	32,902	F-actin-capping protein subunit alpha 1	3.04	3.59	Cytoskeleton organization
CAPZB	30,609	F-actin-capping protein subunit beta isoform 1	3.08	0.54	Cytoskeleton organization
CCT2	57,452	T-complex protein 1 subunit beta isoform 1	−2.78	−2.78	Protein folding
CCT4	57,888	T-complex protein 1 subunit delta isoform A	−3.11	−3.32	Protein folding
CDC42	21,245	Cell division control protein 42 homolog isoform 1 precursor	−3.26	−3.26	Cytoskeleton organization, cell migration
CLTC	191,493	Clathrin heavy chain 1 isoform 1	2.30	2.78	Protein localization
DBN1	71,565	Drebrin isoform B	3.04	4.28	Cytoskeleton organization
ENO1	47,139	Alpha-enolase isoform 1	−3.83	−0.35	Glycolysis
EPB41L2	112,519	Band 4.1-like protein 2 isoform A	2.85	2.85	Cytoskeleton organization, protein localization
EZR	69,370	Ezrin	−0.60	−3.04	Cytoskeleton organization
GNB1	37,353	Guanine nucleotide-binding protein G(I)/G(S)/G(T) subunit beta 1 isoform 1	3.32	3.00	Protein folding
HIST1H1C	21,352	Histone H1.2	3.26	−0.38	DNA/RNA binding
HNRNPF	45,643	Heterogeneous nuclear ribonucleoprotein F	−3.23	−2.90	RNA binding
HNRNPK	50,944	Heterogeneous nuclear ribonucleoprotein K isoform B	2.85	−0.33	Pre-mRNA/mRNA processing
HNRNPU	88,924	Heterogeneous nuclear ribonucleoprotein U isoform B	−2.60	-2.90	DNA/RNA binding
PABPC1	70,626	Polyadenylate-binding protein 1	−2.70	−3.04	Pre-mRNA/mRNA processing
RPS3A	29,926	40S ribosomal protein S3a isoform 1	3.11	0.00	RNA binding
SERPINH1	46,411	Serpin H1 precursor	−2.90	−3.42	Collagen biosynthetic process
SUMO3	11,630	Small ubiquitin-related modifier 3 isoform 1 precursor	3.53	0.00	Protein SUMOylation, DNA binding, protein localization
TKT	67,835	Transketolase isoform 1	−2.70	−2.70	Glyceraldehyde-3-phosphate biosynthetic process
TMEM109	26,194	Transmembrane protein 109 precursor	−3.18	−3.18	Response to DNA damage
TPM1	32,797	Tropomyosin alpha 1 chain isoform Tpm1.3sm	3.38	3.08	Cytoskeleton organization
VASP	39,805	Vasodilator-stimulated phosphoprotein	2.95	2.95	Cytoskeleton organization
VCP	89,266	Transitional endoplasmic reticulum ATPase	2.60	0.00	DNA repair, endoplasmic reticulum unfolded protein response
YBX1	35,903	Nuclease-sensitive element-binding protein 1	−3.08	−0.34	RNA stabilization, mRNA splicing, DNA repair

**FIG 5:**
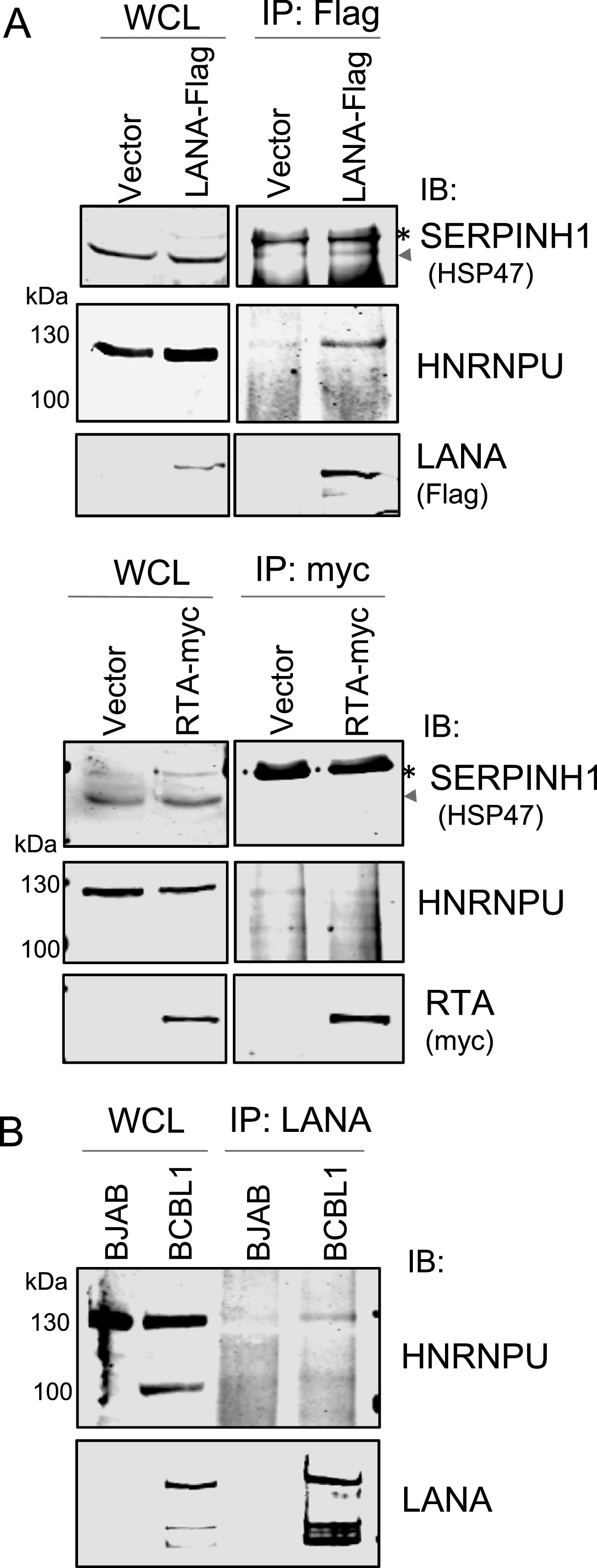
Validation of representative cellular proteins associated with LANA^SIM^. (A) LANA association with SERPINH1 and HNRNPU. The whole-cell lysates (WCL) from HEK293 cells expressing LANA-Flag, RTA-myc, or the vector alone at 48 h posttransfection were subjected to direct immunoblotting (IB) or coimmunoprecipitation (IP) followed by immunoblotting with the indicated antibodies. The asterisk indicates a nonspecific band. (B) Endogenous HNRNPU interacts with LANA in PEL cells. KSHV-positive BCBL1 PEL cells and negative BJAB B lymphoma cells were subjected to direct immunoblotting or coimmunoprecipitation followed by immunoblotting with the indicated antibodies.

**FIG 6:**
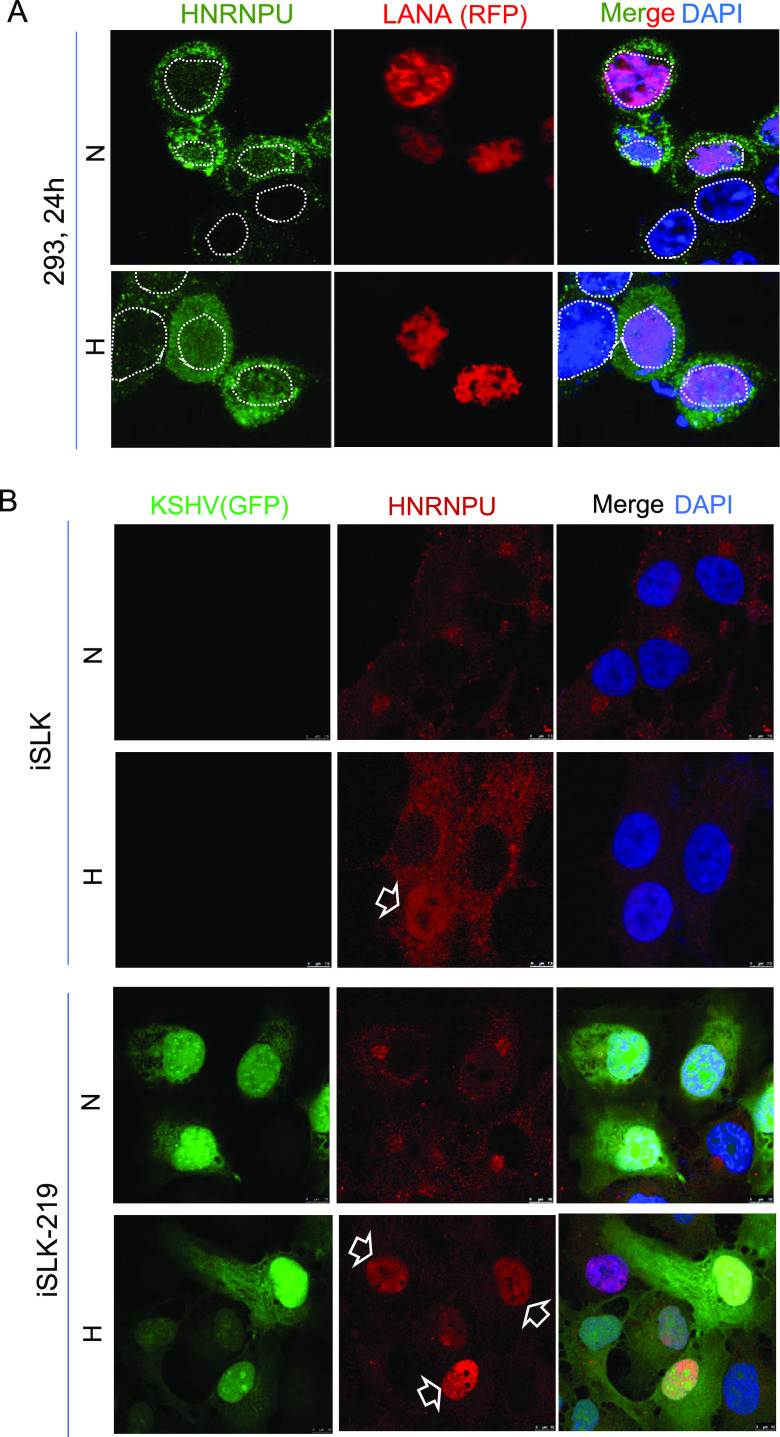
Expression of HNRNPU is enhanced by KSHV latent infection and relocalizes to the nuclear compartment in hypoxia. (A) Expression of HNRNPU is upregulated by LANA and relocalizes to the nuclear compartment in hypoxia. HEK293 cells expressing RFP-LANA were treated with normoxia (N) or hypoxia (H) for 24 h after 24 h of transfection, followed by immunofluorescence assays with antibodies against HNRNPU. Nuclei are stained with DAPI. (B) Expression of HNRNPU is enhanced by KSHV latent infection and relocalizes to the nuclear compartment in hypoxia. The iSLK cell line and its derived iSLK.219 cells (with green fluorescent protein [GFP]-KSHV latent infection) were individually treated with normoxia or hypoxia for 24 h after 24 h of transfection, followed by immunofluorescence assays with antibodies against HNRNPU. Nuclei are stained with DAPI. Arrows indicate the nuclear localization of HNRNPU.

To address whether there are some identified proteins that overlap between the LANA^SIM^-associated proteome profile in hypoxia and the GST-SIM-associated proteome profile reported previously (15), we aligned each protein within the LANA^SIM^ and GST-SIM profiles. Surprisingly, there were only two proteins, namely, SERPINH1 and HNRNPU, that were identified in both the LANA^SIM^ and GST-SIM profiles, while most of the other proteins were exclusive for each of their independent profiles (Fig. 4D). To further validate the potential association of SERPINH1 and HNRNPU with LANA^SIM^ in response to hypoxia, we performed coimmunoprecipitation analyses by overexpressing LANA or RTA in HEK293 cells. The results showed that SERPINH1 and HNRNPU, but not RTA, associated with LANA to some extent (Fig. 5A). In view of the fact that LANA has a higher affinity for HNRNPU than SERPINH1, the interaction of endogenous LANA with HNRNPU was further confirmed in PEL cells by coimmunoprecipitation assays with LANA-specific antibodies (Fig. 5B). The results from immunofluorescence assays showed not only that the expression of LANA in HEK293 cells dramatically enhanced the expression levels of the endogenous HNRNPU protein in normoxia but also that hypoxia treatment increased HNRNPU localization with LANA in the nuclear compartment (Fig. 6A). These data provide strong evidence that HNRNPU associates with LANA^SIM^ and responds to hypoxia.

### The SUMOylation of HNRNPU is enhanced by KSHV latent infection and responds to hypoxia.

**FIG 7:**
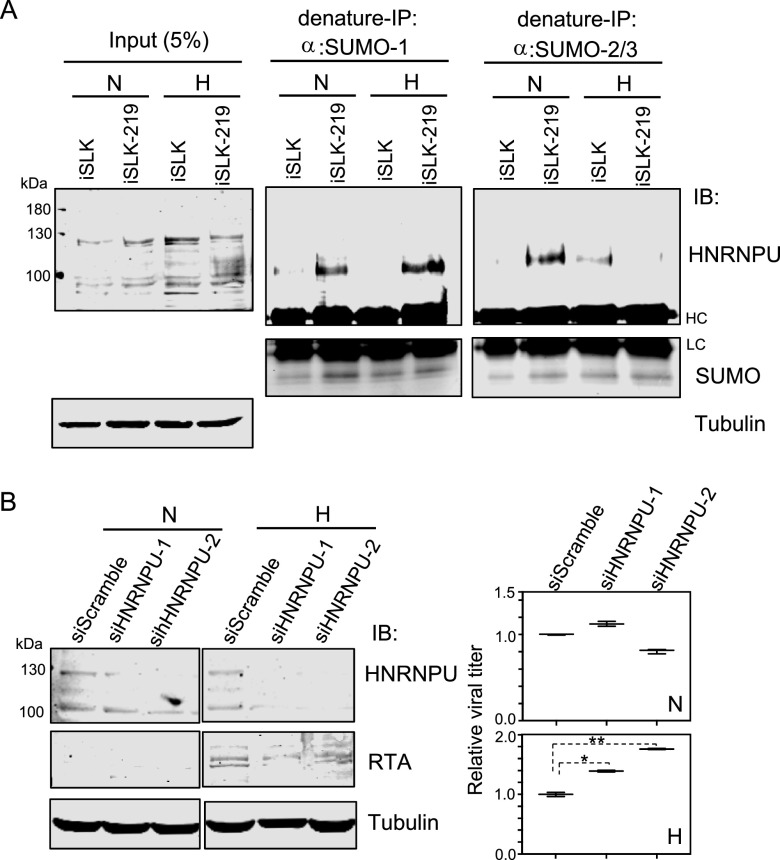
SUMOylation of HNRNPU is enhanced by KSHV latent infection and responds to hypoxia. (A) SUMOylation of HNRNPU is enhanced by KSHV latent infection and responds to hypoxia. Whole-cell lysates (WCL) from the iSLK cell line and its derived iSLK.219 cells with normoxia (N) or hypoxia (H) treatment for 24 h were subjected to direct immunoblotting (IB) or denatured immunoprecipitation (IP) followed by immunoblotting with the indicated antibodies. HC, heavy chain; LC, light chain. (B) HNRNPU knockdown enhances KSHV virion production in hypoxia. KSHV latently infected iSLK.219 cells individually transfected with small interfering RNA against HNRNPU (siHNRNPU) or the scramble control were subjected to normoxia or hypoxia treatment for 24 h at 24 h posttransfection, followed by lysis and immunoblotting with the indicated antibodies. In the right panels, the supernatant of the cell culture medium was harvested and subjected to quantitation of KSHV virion production.

To further determine the role of HNRNPU in KSHV latently infected cells and in response to hypoxia, we performed immunofluorescence assays to examine the localization of HNRNPU in the iSLK cell line and its derived iSLK.219 cells with KSHV latent infection under normoxia or hypoxia conditions. Consistent with the observations from overexpression with exogenous LANA, the results showed that both the expression and nuclear location of HNRNPU are enhanced by KSHV latent infection in iSLK.219 cells under normoxia conditions, and the nuclear localization of HNRNPU is further induced by hypoxic stress (Fig. 6B). In view of the fact that HNRNPU associates with LANA^SIM^, we attempted to examine whether HNRNPU undergoes SUMOylation in response to KSHV latent infection and hypoxia. The results from denatured immunoprecipitation (IP) assays showed that HNRNPU was dramatically SUMOylated by SUMO1 and SUMO2/3 in iSLK cells with KSHV latent infection (iSLK.219) under normoxic conditions, while the SUMO2/3- but not the SUMO1-modified form of HNRNPU was inhibited by hypoxia (Fig. 7A), indicating that SUMO2/3-modified HNRNPU induced by KSHV infection is involved in the response to hypoxia.

To further investigate the biological role of HNRNPU in the regulation of KSHV reactivation of lytic replication, we knocked down the expression of HNRNPU in iSLK.219 cells by the introduction of small interfering RNA and looked at the expression level of RTA (the master regulator of lytic replication) and virion production in the culture medium in the presence and absence of hypoxia treatment. The results showed that inhibition of HNRNPU expression did not significantly impair RTA expression or virion production in normoxia (Fig. 7B, left). In contrast, although the expression of RTA did not have a significant effect, inhibition of HNRNPU greatly increased virion production induced by hypoxia (Fig. 7B, right). These data suggest that HNRNPU is involved in the regulation of the KSHV life cycle and responds to hypoxia.

## DISCUSSION

To globally address the biological role of the SIM of LANA in KSHV-mediated latent infection, we report the systematic analysis of cellular proteins associated with the SIM of LANA in hypoxia by generating HUVECs and HEK293 cells stably expressing wild-type LANA (LANA^WT^) and its SIM-deleted mutant (LANA^dSIM^). Specifically, we found that molecules involved in the regulation of two major cellular pathways, which include cytoskeleton organization and DNA/RNA binding and processing pathways, are significantly targeted by LANA^SIM^ in response to hypoxia.

It is worth mentioning that although our previous studies revealed a proteomic profile of 155 LANA^SIM^-associated proteins when we used a recombinant protein of GST fused with LANA^SIM^ (the region from amino acids 240 to 330 of LANA) as bait to pull down the interacting proteins from nuclear extracts of KSHV-positive PEL cells *in vitro* (15), it remains uncertain as to whether the LANA^SIM^-associated proteins play functional roles under physiological conditions in KSHV-infected cells. To further identify the proteomic profile of the LANA^SIM^-interacting proteins with the intact SIM of LANA, we selected two types of endothelial cells, HUVECs (one of two cell types mainly infected by KSHV) and HEK293 kidney cells (as a parallel control), to stably express wild-type LANA or its SIM-deleted mutant, followed by mass spectrometry analysis after coimmunoprecipitation. To increase the specificity and accuracy of the identified cellular proteins associated with LANA^SIM^, the vector-alone group along with the SIM-deleted mutant group were used as controls to rule out the nonspecific background of protein-protein interactions. Unexpectedly, we obtained two major proteomic profiles of identified LANA^SIM^-interacting cellular proteins from HEK293 cells and HUVECs that were significantly different in hypoxia, although there were 28 proteins identified in both types of cells. To address the potential possibility of a difference because of the two cell lines, we analyzed the cell cycle, expression level of LANA, cell viability, and proliferation of stable cell lines in hypoxia. Interestingly, we found that HUVECs stably expressing LANA^dSIM^ were more sensitive to hypoxia than HUVECs stably expressing LANA^WT^. Cell apoptosis was also increased when cells were treated in hypoxia (data not shown), which could be an explanation for why cellular proteins interacting with LANA^WT^ were increased in hypoxia while they were decreased in the LANA^dSIM^ group. This evidence indicates that the SIM plays an important role in the response to hypoxic stress for inhibition of cell apoptosis. In contrast, among 28 LANA^SIM^-associated proteins in hypoxia, only 2 molecules, SERPINH1 and HNRNPU, appear in the profile of 155 LANA^SIM^-associated proteins using the GST-SIM recombinant protein as bait. This suggests that both SERPINH1 and HNRNPU are commonly and significantly targeted by LANA^SIM^ in these two different screening systems. Further investigation revealed that SUMOylation of HNRNPU is greatly enhanced by KSHV latent infection, while only the SUMO2/3- and not the SUMO1-modified form of HNRNPU induced by KSHV responds to hypoxia. Knockdown of HNRNPU increased KSHV virion production induced by hypoxia, indicating that SUMO2/3-modified HNRNPU is involved in the regulation of the hypoxia response and KSHV latent-lytic life cycle control. The fact that SUMO3, as one of 28 LANA^SIM^-associated proteins in both HUVECs and HEK293 cells, was consistently identified, and there are higher percentages of identified proteins containing at least one SIM in the LANA^SIM^ group than in the LANA^dSIM^ group, further supports the results from the LANA^SIM^-associated screening system.

To analyze the universal biological function of LANA^SIM^ in a variety of cell lines, we chose proteins with similar changing trends and that were identical between HUVECs and HEK293 cells after the SIM was deleted in hypoxia. Among the 28 target proteins, ACTB, ACTN1, ACTN4, CAPZA1 CAPZB, DBN1, TPM1, and VASP are associated with building and maintaining the cytoskeleton. Supporting these studies, a recent report showed that SUMO is covalently attached to components of each of the four major cytoskeletal networks, including microtubule-associated proteins, septins, and intermediate filaments (18, 19). For example, ACTN4 is involved in the formation of F-actin and the regulation of cell migration and tumor invasion (20, 21), and DBN1 plays a role in cell migration and actin polymerization (22). In addition, some groups discovered that hypoxia treatment also induced actin cytoskeleton remodeling by regulating the binding of CAPZA1 to F-actin (23, 24), which further supports our observation that the SIM of LANA participates in the regulation of cell migration and tumor invasion in hypoxia.

In summary, we have identified a proteomic profile of host proteins related to the SIM of LANA in hypoxia through coimmunoprecipitation combined with MS analysis and found that the main cellular pathways regulated by LANA^SIM^ are different when cells are cultured in normoxia or hypoxia. Based on the analysis of the intersecting proteins of both HUVECs and HEK293 cells, we revealed that the SIM of LANA is involved in two major pathways, including cytoskeleton organization and DNA/RNA binding and processing pathways, in hypoxia, for which further experiments for protein validation and functional analysis are required.

## MATERIALS AND METHODS

### Cell culture.

HEK293, HEK293T, iSLK (1 μg/ml puromycin and 250 μg/ml G418) (a gift from Shou-Jiang Gao at the University of Southern California), and iSLK.219 (1.1 mg/ml hygromycin, 250 μg/ml G418, and 1 μg/ml puromycin) (25) cells were maintained in Dulbecco’s modified Eagle’s medium (DMEM) supplemented with 10% fetal bovine serum (FBS), 1% penicillin (Sangon Biotech Inc., Shanghai, People’s Republic of China), and streptomycin (BBI Life Sciences, Shanghai, People’s Republic of China). HUVECs, BJAB cells, and BCBL1 cells were cultured with RPMI 1640 medium containing 10% FBS. All cell lines were incubated at 37°C in a humidified environmental incubator with 5% CO_2_. For hypoxia treatment, cell lines were incubated at 37°C in a humidified environmental incubator with 5% CO_2_ and 0.2% O_2_ in a BioSpherix hypoxic culture system.

### DNA construction.

The plasmids pA3M-RTA, pA3F-LANA^WT^, and pLVX-Puro-YFP-LANA^WT^-Flag expressing the full-length LANA fusion with a Flag tag were described previously (14, 26, 27). The plasmids pA3F-LANA^dSIM^ and pLVX-Puro-YFP-LANA^dSIM^-Flag carrying the SIM-deleted mutant of LANA were constructed by PCR-directed site mutation.

### Establishment of LANA stable expression cell lines.

The pLVX-Puro vectors carrying LANA^WT^ and LANA^dSIM^ cDNAs were cotransfected with lentivirus packaging plasmids into HEK293T cells for 48 h to generate lentiviruses. For transduction, the packaged lentiviruses were individually added to HUVECs and HEK293 cells and centrifuged for 1 h at 1,500 × *g* with 6 mg/ml Polybrene (catalog number H9268; Sigma, Shanghai, People’s Republic of China). Medium was replaced after the spin, and 2 μg/ml (final concentration) of puromycin was added at 24 h posttransduction. Selection was carried out until colonies were clonal with 100% green fluorescence for transduced cells.

### Protein fractionation and mass spectrometry.

The sodium dodecyl sulfate (SDS)-PAGE gel lanes of YFP (vector), LANA^WT^, and LANA^dSIM^ from stable HEK293 cell or HUVEC lines were individually cut into pieces using a sterile in-house cutting device. Protein standard bands served as a guide for the excision of gel slices of various molecular weight ranges. The protocols for gel slice recycling and protein digestion were performed as described previously (15, 28). Extracted peptides were resuspended in 10 μl of a solution containing 5% acetonitrile and 0.1% trifluoroacetic acid and run on a NanoAcquity ultra high performance liquid coupled two-dimensional linear ion trap electrostatic Orbitrap XL hybrid mass spectrometer equipped with electron transfer dissociation (UPLC/LTQ Orbitrap XL ETD) tandem quantitative mass spectrometer (Thermo Fisher Scientific, Shanghai, People’s Republic of China).

### Protein identification by mass spectrometry and data analysis.

We chose emPAI values (the emPAI value is a quantitative protein value that can predict protein abundance based on the number of detected peptides) to analyze the relative quantitative profiles of nonlabeled proteins (15). Proteins in the YFP group were used as a blank control, and proteins in the LANA groups with emPAI values ≥2 times the values of the control group were selected for analysis. The data were analyzed with GPS explorer/Analyst QS software (15) and searched with Mascot software (Matrix Science Ltd.) against the National Center for Biotechnology Information (NCBI) database. Pathway and function annotation information was downloaded from MSigDB database v5.0, and Venn diagrams were created with Venny2.1. Enrichment analysis was fulfilled between the core and extended networks and the currently available pathways and functional categories. The most significant pathways and function categories were picked based on the enrichment *P* values. The enrichment *P* value is calculated according to the hypergeometric distribution in this study. We used GPS-SUMO to predict SIMs and SUMOylated sites of selected proteins.

### Immunoprecipitation, denatured immunoprecipitation, and immunoblotting.

Immunoprecipitation (IP), denatured IP, and immunoblot (IB) assays were performed as described previously (14, 29). Briefly, 30 million cells were cultured in normoxia (21% O_2_) and hypoxia (0.2% O_2_) for 48 h. The cells were harvested, washed twice with cold phosphate-buffered saline (PBS), and then lysed in 1 ml radioimmunoprecipitation assay (RIPA) buffer (150 mM NaCl, 50 mM Tris [pH 7.6], 1% NP-40, 2 mM EDTA, and a protease inhibitor cocktail) for 30 min on ice, with brief vortexing every 5 min. The lysates were centrifuged at 14,500 rpm at 4°C for 5 min to remove cell debris. The supernatant was incubated with 5 μg anti-Flag M2 mouse monoclonal antibody (Sigma) at 4°C with rotation overnight. The next day, the supernatants were incubated with 60 μl protein A/G-Sepharose beads (GE Co.) at 4°C with rotation for another 2 h and centrifuged at 3,000 rpm at 4°C for 2 min to remove the supernatant. The precipitates were washed 3 times with 1 ml Tris-buffered saline (TBS) buffer (300 mM NaCl, 20 mM Tris-HCl), resuspended with 50 μl PBS and 10 μl 6× SDS loading buffer, and boiled at 100°C for 8 min. The boiled proteins were separated by SDS-PAGE on a 4 to 15% precast gel (Bio-Rad), and the gel was stained using Coomassie stain. For denatured IP, cells were harvested, directly lysed in ice-cold RIPA buffer with 1% SDS, and boiled for 10 min. Cell debris was removed by centrifugation, and the supernatants were then transferred to a new Eppendorf tube. The supernatant lysates were then precleared, beads were spun out, and the mixture was then incubated with primary antibody overnight. Complexes of proteins of interest were captured with protein A/G-Sepharose beads and boiled in loading buffer. For immunoblotting, protein samples were separated by SDS-PAGE and transferred to a nitrocellulose membrane. The membrane was blocked with 5% nonfat dry milk and probed with the following corresponding antibodies: Flag (M2; Sigma), SERPINH1 (catalog number Ag1301; Proteintech) or HNRNPU (catalog number Ag9151; Proteintech), RTA (4D2), tubulin (catalog number Ag18034; Proteintech), SUMO1 (catalog number Ag0414; Proteintech), and SUMO2/3 (catalog number Ag1778; Proteintech). The interesting proteins in the membrane were scanned and analyzed by using the Odyssey infrared scanner and its software (Li-Cor Biosciences).

### Immunofluorescence.

Immunofluorescence assays were performed as described previously (7). Briefly, HEK293 cells transfected with a plasmid expressing LANA-red fluorescent protein (RFP) were directly grown on sterile coverslips for 48 h, washed with ice-cold PBS once, and fixed in 3% paraformaldehyde for 20 min at room temperature. After fixation, cells were washed three times and permeabilized in PBS containing 0.2% fish skin gelatin (catalog number G-7765; Sigma) and 0.2% Triton X-100 for 5 min, followed by incubation with the specific primary and secondary antibodies. DNA was counterstained with DAPI (4′,6′-diamidino-2-phenylindole), and coverslips were mounted with *p*-phenylenediamine. Cells were visualized with a Leica SP8 confocal microscope.

### RNA interference.

Oligonucleotide sequences against HNRNPU (target sequence 1, 5′-GGCCUGAAGAGAAGAAAGATT-3′; target sequence 2, 5′-GUGGCAAGAACCAGUUUAATT-3′) and a nonspecific scramble control sequence (5′-UUCUCCGAACGUGUCACGUTT-3′) were used. The oligonucleotides were individually transfected into iSLK.219 cells by using the GP-transfect-Mate transfection reagent according to the manufacturer’s protocol (GenePharma, Shanghai, People’s Republic of China). Immunoblot analysis with HNRNPU antibody was individually used to verify the efficiency of RNA interference.

### Quantitation of KSHV virions in the supernatant.

iSLK.219 cells with or without HNRNPU knockdown cells were subjected to hypoxia treatment for 24 h at 37°C with 5% CO_2_. After treatment, the supernatant of the culture medium was collected and filtered through a 0.45-μm filter, and viral particles were spun down at 25,000 rpm for 2 h at 4°C. The concentrated virus was collected and used for virion quantitation by quantitative PCR as described previously (27).

### Statistical analysis.

Data were analyzed using Statistical Package for Social Science (SPSS) software 20.0 (IBM Corp., Armonk, NY) for statistical analysis. All statistical tests were two tailed, and results were considered significant when the *P* value was less than 0.05.
